# CaLMPhosKAN: prediction of general phosphorylation sites in proteins via fusion of codon aware embeddings with amino acid aware embeddings and wavelet-based Kolmogorov–Arnold network

**DOI:** 10.1093/bioinformatics/btaf124

**Published:** 2025-03-21

**Authors:** Pawel Pratyush, Callen Carrier, Suresh Pokharel, Hamid D Ismail, Meenal Chaudhari, Dukka B KC

**Affiliations:** Golisano College of Computing and Information Sciences, Rochester Institute of Technology, Rochester, NY 14623, United States; College of Computing, Michigan Technological University, Houghton, MI 49931, United States; Golisano College of Computing and Information Sciences, Rochester Institute of Technology, Rochester, NY 14623, United States; College of Engineering, North Carolina Agricultural and Technical State University, Greensboro, NC 27411, United States; College of Applied Sciences and Technology, Illinois State University, Normal, IL 61761, United States; Golisano College of Computing and Information Sciences, Rochester Institute of Technology, Rochester, NY 14623, United States

## Abstract

**Motivation:**

The mapping from codon to amino acid is surjective due to codon degeneracy, suggesting that codon space might harbor higher information content. Embeddings from the codon language model have recently demonstrated success in various protein downstream tasks. However, predictive models for residue-level tasks such as phosphorylation sites, arguably the most studied Post-Translational Modification (PTM), and PTM sites prediction in general, have predominantly relied on representations in amino acid space.

**Results:**

We introduce a novel approach for predicting phosphorylation sites by utilizing codon-level information through embeddings from the codon adaptation language model (CaLM), trained on protein-coding DNA sequences. Protein sequences are first reverse-translated into reliable coding sequences by mapping UniProt sequences to their corresponding NCBI reference sequences and extracting the exact coding sequences from their GenBank format using a dynamic programming-based global pairwise alignment. The resulting coding sequences are encoded using the CaLM encoder to generate codon-aware embeddings, which are subsequently integrated with amino acid-aware embeddings obtained from a protein language model, through an early fusion strategy. Next, a window-level representation of the site of interest, retaining the full sequence context, is constructed from the fused embeddings. A ConvBiGRU network extracts feature maps that capture spatiotemporal correlations between proximal residues within the window. This is followed by a prediction head based on a Kolmogorov-Arnold network (KAN) using the derivative of gaussian wavelet transform to generate the inference for the site. The overall model, dubbed CaLMPhosKAN, performs better than the existing approaches across multiple datasets.

**Availability and implementation:**

CaLMPhosKAN is publicly available at https://github.com/KCLabMTU/CaLMPhosKAN.

## 1 Introduction

Protein phosphorylation is a well-studied post-translational modification (PTM) and involves the covalent addition of a phosphate group to the side chain of specific amino acids, typically mediated by kinase enzymes (Que *et al.* 2010). This modification is most commonly observed on the residues of Serine (S), Threonine (T), and Tyrosine (Y), though it also occurs on Aspartic acid, Arginine, Cysteine, and Histidine (Lapek *et al.* 2015, Leijten *et al.* 2022). Phosphorylation serves as a fundamental regulatory mechanism in various cellular processes, including subcellular localization, cell growth and division, protein stability, and signal transduction (Johnson 2009). Dysregulation of phosphorylation, potentially induced by natural toxins or pathogens, can lead to severe diseases such as cancer, Alzheimer’s disease, and heart disease, among others (Ardito *et al.* 2017). Consequently, the identification and understanding of phosphorylation sites are critical for developing new therapeutic strategies and insights into drug design (Nalepa *et al.* 2006, Gyawali *et al.* 2024).

In recent years, there has been a noticeable shift from traditional machine learning-based models to more advanced deep learning-based models for PTM prediction. Machine learning methods such as RFPhos (Ismail *et al.* 2016), NetPhosK (Hjerrild *et al.* 2004), KinasePhos (Ma *et al.* 2023), and GPS (Zhou *et al.* 2004) relied on manually extracted features. While these models provided acceptable prediction capabilities, they were generally outperformed by the more sophisticated deep learning (DL) approaches. DL methods in this field typically utilize learned embeddings or leverage pre-trained language models to enhance prediction accuracy. One of the pioneering deep learning models, Musite (Wang *et al.* 2020), introduced the use of attention-based mechanisms to improve the prediction of phosphorylation sites. Building on this, the same research group developed CapsNet (Wang *et al.* 2022), which used a capsule network architecture. This model not only marginally improved upon its predecessor but also enhanced the interpretability of the predictions through the use of capsules. However, a major limitation of both models was their reliance on isolated protein fragments (peptides) for modeling, which restricted their ability to capture the global context influencing phosphorylation sites. DeepPSP (Guo *et al.* 2021) sought to address this limitation by combining representations from a sequence of 2000 residues (referred to as the global information module) with peptide-level information (the local information module). Most recently, LMPhosSite (Pakhrin *et al.* 2023) further advanced the field by incorporating full-sequence context using a protein language model (pLM) and predicting phosphorylation sites with residue-level embeddings. A detailed literature review of these tools is available in the Supplementary Section S1. Despite these advancements, there remains scope for improving predictive performance. Many of these tools have relied on local peptide-based encoding, and while LMPhosSite used full-sequence context, it was limited to residue-level embeddings. Moreover, all these tools, whether for phosphorylation sites prediction or other PTMs, have primarily used representations derived from amino acid sequences. However, Outeiral and Deane (2024) recently introduced codon adaptation language model (CaLM), a pLM trained on protein-coding DNA sequences, asserting the probable presence of higher information in codon space. Their claim was supported by empirical evidence showing that CaLM outperformed amino acid-based state-of-the-art pLMs, such as ESM (Lin *et al.* 2022), ProtT5(Elnaggar *et al.* 2022), and ProtBERT (Elnaggar *et al.* 2022), in variety of protein-level downstream tasks including melting point prediction, solubility prediction, subcellular localization classification, and function prediction.

In this study, we explore the potential of codon-aware embeddings for general phosphorylation sites prediction, an important residue-level task. We expand the contemporary approaches that rely on amino acid-aware pLMs by integrating embeddings from CaLM, which aims to capture potentially higher information content inherent in the codon space. Specifically, we combine these codon-aware embeddings from CaLM with amino acid-aware embeddings from the ProtTrans encoder to create a bimodal representation of the full sequence. A window-level representation is then constructed around each target site to extract spatiotemporal features of the surrounding residues, which are subsequently learned by a wavelet-based Kolmogorov–Arnold network (Wav-KAN). Our model, termed CaLMPhosKAN, outperforms existing state-of-the-art predictors, demonstrating that integrating codon-based embeddings can enhance general phosphosites prediction. Additionally, we present the robustness of our model in predicting phosphorylation sites within disordered regions of proteins. To the best of our knowledge, this is the first work to utilize codon-aware embeddings in the PTM prediction problem and residue-level task in general.

## 2 Materials and methods

### 2.1 Datasets construction

#### 2.1.1 Phosphosite datasets

The *primary* dataset used to build the CaLMPhosKAN model was curated by the DeepPSP group (Guo *et al.* 2021) comprising experimentally identified phosphorylated sites on serine (S), threonine (T), and tyrosine (Y) residues. The sequences were subjected to homology reduction using CD-HIT with a similarity cut-off of 0.5, following the original study. Additionally, we conducted ablation experiments using cut-offs ranging from 0.3 to 0.5 (in increments of 0.1) and found 0.5 to be optimal, corroborating the value used in DeepPSP. The corresponding results are presented in Supplementary Section S2. For labeling, experimentally annotated S, T, and Y residues were designated as phosphorylated sites (P-sites), whereas the remaining unannotated S, T, and Y residues within the same sequences were considered non-phosphorylated (NP-sites). Following this, the dataset was randomly partitioned into training and test sets in a 9:1 ratio based on protein IDs instead of sites, ensuring no overlap of sequences between the sets.

To further assess the generalizability of the CaLMPhosKAN model, we tested its performance on two additional datasets. The first was a test set curated from human *A549* cells infected with the SARS-CoV-2 virus, derived from the literature (Lv *et al.* 2021), consisting of experimentally identified P-sites on S and T residues. The second consisted of training and test datasets for S and T residues from the organism *Chlamydomonas reinhardtii* (*C. reinhardtii*), adopted from the literature (Thapa *et al.* 2021). The preprocessing steps of these additional datasets are detailed in Supplementary Section S3.

Given the structural and functional similarities between serine and threonine residues, we combined their datasets (Swingle and Honkanen 2019, Pakhrin *et al.* 2023). In contrast, tyrosine residues are subject to phosphorylation through distinct enzymatic processes (Gutzler and Perepichka 2013, Pakhrin *et al.* 2023) and hence, we maintained a separate dataset for *Y* sites, distinct from the combined S/T (or *S + T*) dataset.

#### 2.1.2 Reverse translation procedure

**Figure 1.**
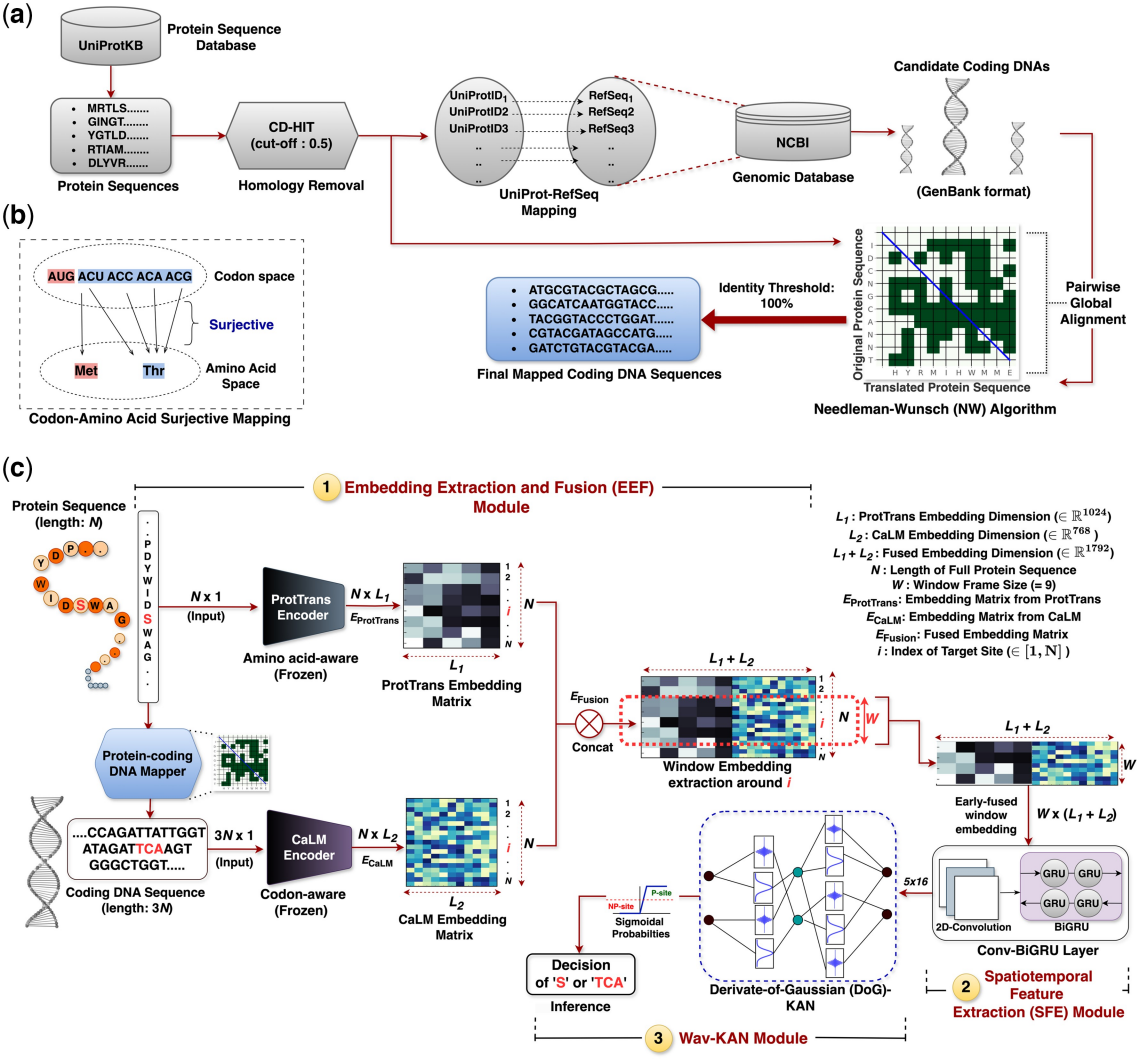
(a) A high-level overview of the dataset preparation and the mapping procedure for obtaining protein-coding DNAs. (b) Illustrating the nature of codon alphabets mapping to amino acid alphabets, showing a surjective mapping. For instance, the amino acid Methionine (Met, *M*) is mapped by a single codon *AUG*, while Threonine (Thr, *T*) can be mapped by at most four codons: *ACU*, *ACC*, *ACA*, and *ACG*. (c) The architecture of CaLMPhosKAN depicting the three modules—(1) Embedding Extraction and Fusion (*EEF*) Module, (2) Spatiotemporal Feature Extraction (*SFE*) Module, and (3) Wav-KAN Module. The site of interrogation in the protein sequence is “S” (at index *i*) or “TCA” (at index 3*i*) in the corresponding coding DNA, and is highlighted in bold red. The input is the full-length protein sequence containing the site, provided to the *Embedding Extraction and Fusion Module*, and the output is the logit of the target site (S/TCA) received from the *Wav-KAN Module*.

The subsequent step involved “reverse translation,” where the protein sequences were translated to their corresponding coding DNA sequences, a process characterized by non-injective mapping (or surjective mapping if considered in the reverse direction, see Fig. 1b). We initiated this by generating protein-coding sequences through the mapping of UniProt identifiers to NCBI nucleotide reference sequences (RefSeq). These RefSeqs are curated to be non-redundant and reliable representations of nucleotide sequences. We used the accession numbers of the identified RefSeqs to retrieve their corresponding candidate coding sequences from the NCBI Nucleotide database (Benson *et al.* 2013) (*timestamp*: May 2024), specifically in GenBank format. This format provides comprehensive details, including the coding sequences and the translated protein product. To ensure the accuracy of the coding sequences as the correct coding sequence for each mapped UniProt protein, we performed a pairwise global alignment using the Needleman–Wunsch (NW) algorithm (Needleman and Wunsch 1970) between the translated protein sequence from the GenBank format and the original UniProt sequence. The NW algorithm uses dynamic programming to find the optimal global alignment of the two sequences by iteratively dividing the entire alignment process into sub-alignments. The identity percent score for each alignment was computed. For inclusion in our dataset, we selected only those coding sequences where the alignment demonstrated 100% identity with the UniProt protein sequence. Moreover, proteins without corresponding RefSeqs were discarded. Details of the changes in the number of sites before and after the translation process of the DeepPSP dataset can be found in Supplementary Section S4. Finally, the termination codons (stop codons) *TAA* (Ochre), *TAG* (Amber), and *TGA* (Opal/Umber) were removed from each coding sequence, resulting in the coding DNA sequence length being exactly three times the length of the corresponding protein sequence, aligning with the triplet nature of the genetic code. The high-level overview of the whole procedure is depicted in Fig. 1a. Table 1 summarizes the final processed data (following reverse translation but before balancing) for the three datasets, including information about the count of P-sites and NP-sites, the CD-HIT threshold applied, and the NP to P ratio.

**Table 1.** Distribution of train and independent test sets for the three datasets and target residues (prior to balancing).

Set	Target residue	Train			Independent test			CD-HIT threshold
		No. of P-sites	No. of NP-sites	Ratio (NP:P)	No. of P-sites	No. of NP-sites	Ratio (NP:P)	
*Primary* ^a^	S + T	154 220	800 329	5.19:1	16 964	85 057	5.01:1	0.5
	Y	27 077	123 918	4.57:1	3054	13 347	4.37:1	0.5
*Chlamydomonas reinhardtii*	S + T	17 345	460 015	26.52:1	4338	115 005	26.51:1	N/A
*A549*	S + T	N/A	N/A	N/A	1144	1049	0.92:1	0.3

a The adopted DeepPSP dataset, after reverse translation, is referred to as the *primary* dataset. The number of sites in both the *S + T* and *Y* sets in this dataset differs from those reported in the DeepPSP paper due to the loss of some sequences during the translation process.

In the final step, the training sets of *primary* (*S + T* and *Y*) and *C. reinhardtii* were balanced using random undersampling of the negative set to avoid biased modeling. Additionally, the independent test set of *C. reinhardtii* were also balanced to match the preprocessing done by the source literature (Thapa *et al.* 2021) and ensure a fair comparison. Reflecting on many works in phosphorylation prediction (Hjerrild *et al.* 2004, Zhou *et al.* 2004, Wang *et al.* 2020, Guo *et al.* 2021, Ma *et al.* 2023, Pakhrin *et al.* 2023) (also refer to Supplementary Section S5), we developed two distinct models: one for the combined *S + T* residues and another specifically for *Y* residues.

### 2.2 Protein embeddings

The highly degenerate nature of codons leads to a surjective mapping from multiple codons to a single amino acid, with most amino acids being encoded by up to six different codons (see Fig. 1b). This indicates that a sequence represented at the codon level might contain as much, if not more, information than the same sequence at the amino acid level. Furthermore, it is known that codon usage influences synthesis rates and protein folding (Bahiri-Elitzur and Tuller 2021, Moss *et al.* 2024). This, in turn, affects the accessibility of residues to kinases and phosphatases for phosphorylation, and also shapes the structural context of proteins, determining the spatial arrangement of P-sites, which can impact how phosphorylation affects protein function (Swingle and Honkanen 2019).

To harness the potential of codon space, we adopt a bimodal representation of input sequences, combining amino acid-level with codon-level informational modes. This approach generates a richer sequence representation by combining contextualized embeddings from both domains, each produced by protein language models (pLMs) pre-trained on their respective modalities. Codon-level embeddings, derived from a pLM trained on coding DNA sequences, are designed to capture the codon usage patterns and synonymous codon preferences that influence translation efficiency and accuracy. Conversely, amino acid-level embeddings, obtained from a pLM trained on protein sequences, primarily reflect the functional potentials of proteins based on their amino acid composition. A detailed explanation of these embeddings is provided below.

#### 2.2.1 Codon-aware embeddings

The coding DNA sequences are encoded using a specialized codon-aware pLM called CaLM (Outeiral and Deane 2024). Built on the Evolutionary Sequence Modeling (ESM) framework, CaLM utilizes an architecture comprising 12 encoder layers (each with 12 attention heads) and a prediction head, amounting to 86 million parameters in total. This model undergoes pretraining using a masked language modeling denoising objective on a dataset of approximately nine million non-redundant coding sequences derived from whole-genome sequencing.

Prior to encoding input coding sequences, the sequences are tokenized into integer tokens that map to the 64 possible codons “words”, along with special tokens. The special tokens <CLS> (classification) and <EOS> (end-of-sequence) are added at the start and end of the sequence, respectively. The tokenized sequence can be expressed as $T=\{<\mathrm{CLS}>,t_{1},t_{2},\ldots ,t_{N},<\mathrm{EOS}>\}$, where $t_{i}$ represents the codon token for position i in the sequence. The vectorized tokens are then fed into the encoder, and the last hidden state (also called embeddings) is extracted. For an input coding sequence of length 3 *N*, corresponding to a protein sequence of length N, the last hidden state produced by the model is an embedding matrix of dimension $H\in R^{(N+2)\times L}$, where L=768 is the embedding dimension. After removing the embeddings corresponding to the <CLS> and <EOS> tokens, the final representation is given by $E\in R^{N\times L}$. It is noteworthy that, despite the input coding sequence being three times the length of the corresponding protein sequence, the resulting embedding matrix is aligned with the length of the protein sequence. This matrix, $E\in R^{N\times L}$, serves as input representation for the codon space for downstream learning.

#### 2.2.2 Amino acid-aware embeddings

Amino acid-aware embeddings are derived from a pLM trained on a large corpus of protein sequences. In this work, we utilize a ProtTrans family model called ProtT5 (Elnaggar *et al.* 2022), a prominent pLM established for its high performance in various protein downstream tasks (Chandra *et al.* 2023), including PTM prediction (Pakhrin *et al.* 2023, Pokharel *et al.* 2023, Pratyush *et al.* 2024). To ensure a comprehensive evaluation, we also explored an alternative competitive pLM, ESM-2 (esm2_t36_3B_UR50D), and conducted a comparative analysis, which can be found in Supplementary Section S6. The results indicate that ESM-2 performs on par with ProtT5, consistent with the findings of Jahn *et al.* (2024), which suggest that the choice of pLM for a particular modality is not a decisive factor in overall performance.

ProtT5 is built on the T5 (Text-to-Text Transfer Transformer) (Raffel *et al.* 2020) architecture and has been trained using an MLM denoising objective on the UniRef50 (UniProt Reference Clusters, encompassing 45 million protein sequences) database. The model comprises a 24-layer encoder-decoder architecture (each with 32 attention heads) and contains approximately 2.8 billion learnable parameters. For this work, we used the pre-trained encoder component of ProtT5 to extract embeddings from the input protein sequences.

For a given protein sequence of length N, each amino acid is converted into integer tokens that map to a vocabulary of 21 canonical amino acids plus a special <EOS> token. Non-canonical/rare amino acids such as “U,” “Z,” “O,” and “B” are mapped to a pseudo-amino acid represented as “X.” The tokenized sequence can be expressed as $T=\{t_{1},t_{2},\ldots ,t_{N},<\mathrm{EOS}>\}$, where $t_{i}$ represents the token for position i in the sequence. The sequence is then processed through the encoder’s attention stack of the ProtT5 model. From this, we extract the last hidden state, $H\in R^{(N+1)\times L}$, where L=1024. As a result, with an input protein sequence of length N, ProtT5 produces amino-acid context-aware embeddings with dimensions (N+1)×1024. The embedding vector corresponding to the <EOS> token is discarded, leaving a final representation of $E\in R^{N\times 1024}$, which is used for downstream learning.

### 2.3 CaLMPhosKAN architecture

The complete schematic of CaLMPhosKAN is depicted in Fig. 1c. We can see that the architecture of CaLMPhosKAN is structured into three interconnected modules. The “Embedding Extraction and Fusion (*EEF*) Module” generates and integrates codon-aware and amino acid-aware window-level embeddings from the pLMs. The “Spatiotemporal Feature Extraction (*SFE*) Module” then extracts feature maps that capture spatiotemporal correlations within local windows around the site-of-interest. Finally, the “Wav-KAN Module” uses a wavelet-induced KAN model to classify these features into P-sites and NP-sites. Note that while the overall architecture remains consistent for *S + T* and *Y* datasets, the model implementation differs only in the Wav-KAN configuration, a detail that will be discussed later. An elaborated explanation of these three modules is presented below.

#### 2.3.1 Embedding extraction and fusion (EEF) module

The *EEF* module integrates multiple components: encoders, a mapper, an early fusion mechanism, and a window-level embedding extractor. Initially, the full protein sequence of length N is processed using the ProtTrans encoder, which transforms the sequence into an embedding matrix of dimension $N\times L_{1}$, where $L_{1}=1024$. Concurrently, the mapper reverse-translates the N-length protein sequence into its corresponding 3 *N*-length coding DNA sequence (*via* procedure detailed in Section 2.1.2). The translated coding sequence is then embedded using the CaLM encoder, resulting in an embedding matrix of size $N\times L_{2}$, where $L_{2}=768$. For the fusion step, the embedding matrix from the ProtTrans encoder $E_{\text{ProtTrans}}\in R^{N\times L_{1}}$ and the embedding matrix from the CaLM encoder $E_{\text{CaLM}}\in R^{N\times L_{2}}$ are concatenated horizontally. This early fusion can be expressed as $E_{\text{Fusion}}=(E_{\text{ProtTrans}}|E_{\text{CaLM}}$), resulting in an embedding matrix of size $N\times (L_{1}+L_{2})$, where $L_{1}+L_{2}=1792$.

To represent a site-of-interest *k*, where *k*∈{S,T,Y} within the sequence, we define a window frame of size W (=2n+1,n≥0,andW≤N) centered around the token corresponding to *k*. This window includes the range [*k*-θ, *k*+θ], where $\theta =\left\lfloor \frac{W}{2}\right\rfloor$ represents the number of upstream and downstream residues, with the site-of-interest *k* positioned at $\left\lfloor \frac{W}{2}\right\rfloor +1$. The embeddings $E_{\text{Fusion}}$ confined to residues within this window frame, constituting a W×1792-dimensional matrix, constitute the window-level representation of site-of-interest *k*. Based on cross-validation experiments and computational efficiency, the optimal value of W was determined to be 9 (i.e. four residues flanking on each side with the fifth residue at the center as the site-of-interest). Notably, all previous predictors compared in this study typically used larger windows (>9) and often relied on isolated sequence fragments to build their models. A key limitation of such an approach is that it may fail to capture the global context influencing the target site. In contrast, we use window-level embeddings attended to residues within the defined window frame, however, each per-residue embedding within this window is generated with awareness of the full sequence context, allowing for global information retention. This ensures that even while focusing on a localized region around the target site, the model preserves and incorporates global sequence-level information, leading to more contextually informed predictions.

#### 2.3.2 Spatiotemporal feature extraction (SFE) module

Kinases, which catalyze the phosphorylation process, often recognize specific sequences or motifs near the phosphorylation sites. Additionally, the neighboring residues can induce conformational changes that either expose or hide potential phosphorylation sites, impacting their accessibility (Subhadarshini *et al.* 2024). To capture these intricate interactions, we use window-level feature extraction in this module to leverage correlations among neighboring residues within a specified window frame around the site-of-interest.

To extract spatial correlations, we utilize a 2D-convolutional layer. The input to this layer is a $W\times (L_{1}+L_{2})$ dimensional matrix from the *EEF* module, where W=9 and $L_{1}+L_{2}=1792$. The layer applies 16 kernels of size 5×5, each operating on a single channel, producing 16 feature maps of dimension 5×1788, corresponding to the window frame. The convolution operation used in this layer can be summarized as F=σ(K∗X+b), where F is the output feature maps, K is the kernel, X is the input matrix comprising window embeddings, b is the bias, and σ(·) is the ReLU activation function. Following the spatial feature extraction, a Bidirectional Gated Recurrent Unit (BiGRU) layer is used with eight units to capture the sequential context within the window frame. The BiGRU processes the feature maps obtained from 2D-CNN bidirectionally, with the forward and backward hidden states given by ${\vec{h}}_{t}=\text{GRU}(F_{t},{\vec{h}}_{t-1})$ and ${\overset{\leftarrow }{h}}_{t}=\text{GRU}(F_{t},{\overset{\leftarrow }{h}}_{t+1})$, respectively. Here, $F_{t}$ represents the feature vector at time t. The final output is a concatenation of these hidden states, $H_{t}={\vec{h}}_{t}|{\overset{\leftarrow }{h}}_{t}$, which encodes bidirectional information, enabling the model to learn dependencies from both upstream and downstream residues relative to the target site. The feature maps output by the overall ConvBiGRU layer has a dimension of 5×16, which serves as an input to the classification module (Wav-KAN), described in Section 2.3.3.

#### 2.3.3 Wav-KAN module

The Wav-KAN module incorporates a prediction head based on the Wav-KAN (Wavelet Kolmogorov Arnold Network) (Bozorgasl and Chen 2024) tasked with rendering the final classification inference. The input to this module is feature maps of 5×16 dimension obtained from the ConvBiGRU network of the *SFE* module, which is flattened into an 80×1 vector before being passed to the Wav-KAN network. Two distinct Wav-KAN models are tailored for the target residue-specific datasets: one for the *S + T* and another for the *Y* datasets. The model designed for the *S + T* dataset comprises two hidden layers, with 128 and 32 nodes, respectively. In contrast, the model for the *Y* dataset is relatively simpler, utilizing a single hidden layer with 24 nodes. A batch normalization layer precedes each hidden layer in both *S + T* and *Y* models.

Unlike traditional Multi-Layer Perceptrons (MLPs) that use fixed activation functions and linear weights at nodes, the KAN architecture utilizes learnable univariate functions on each edge, which are then aggregated across the nodes of subsequent layers. Refer to Supplementary Section S7 for empirical comparison between MLP and KAN using 10-fold cross-validation. Furthermore, the implemented Wav-KAN in this work is a variation of KAN (Liu *et al.* 2024), chosen for its ability to enhance performance and reduce training time by incorporating wavelet transformation functions as adaptive activation functions. These wavelets transform input from each node along the model’s edges through a defined parameterized function called the “mother wavelet.” Wav-KAN supports multiple mother wavelet functions, including both Continuous Wavelet Transform (CWT) and Discrete Wavelet Transform (DWT). For this work, we selected the Derivative of Gaussian (dubbed “DoG”) wavelet function based on its performance in 10-fold cross-validation (refer to Section 3.1). The DoG wavelet can be defined by Equation 1.

(1) $$\psi (t)=-\frac{d}{dt}\left(e^{-t^{2}/2}\right)=t\cdot e^{-t^{2}/2},$$

where ψ(t) represents the wavelet function dependent on the time variable t, derived as the first-order derivative of the gaussian function $e^{-t^{2}/2}$. This function is further trainable, as given in Equation (2) below:

(2) $$\psi_{\;\text{exp}\;}(t)=\omega \cdot \psi (t),$$

where ω serves as a learnable coefficient for the mother wavelet function, enabling fine-tuning of the wavelet shape during backpropagation.

Following the hidden layers, the output passes through a single neuron equipped with a sigmoid activation function, which converts the logit (z) into a probability (p) using the formula $p=\frac{1}{1+e^{-z}}$, where p∈[0,1]. The probability value is used to make the prediction inference, determining whether the target residue (marked as “S” in the protein sequence and “TCA” codon in the corresponding coding DNA sequence in Fig. 1) belongs to a P or NP site. A detailed architectural description of the ConvBiGRU with Wav-KAN integration is provided in Supplementary Section S8, while an ablation study evaluating alternative network architectures can be found in Supplementary Section S6.

### 2.4 Model training and evaluation protocol

In the proposed architecture, the pLM encoders CaLM and ProtTrans are frozen during training, while the downstream models, ConvBiGRU and KAN, are optimized to minimize the binary-cross entropy with logits (BCEwithLogits) loss function using the Adam optimizer. Training is conducted in a mixed precision floating point (utilizing both 16-bit and 32-bit operations) to improve computational efficiency and reduce memory usage. The loss is dynamically scaled during backpropagation using Pytorch’s GradScaler to ensure the gradients are sufficiently large to avoid underflow when using 16-bit precision. An adaptive learning rate of 9e-4 is chosen with decay rates of 0.9 for the first moment and 0.999 for the second moment, and a batch size of 1024 (Guo *et al.* 2021). The optimization of hyperparameters is performed using stratified 10-fold cross-validation on the training set, ensuring that proteins in each training fold are mutually exclusive with those in the corresponding validation set. Early stopping is used to avoid overfitting, and accuracy/loss curves (see sample curves in Supplementary Section S9) are carefully monitored in each fold. All models are implemented in a PyTorch environment using an NVIDIA A100-SXM4-80GB GPU.

Model evaluation is conducted using five performance metrics consistent with existing works: Matthews Correlation Coefficient (MCC), Precision (PRE), Recall (REC) or Sensitivity (SN), F1, and Area Under Curve (AUC). Due to the high degree of imbalance in the *primary* independent test sets, we use the weighted F1-score, i.e. F1_weighted_ (F1_wt_), and the Area Under the Precision-Recall Curve (AUPR) instead of the Area Under the Receiver Operating Characteristic (AUROC) (Saito and Rehmsmeier 2015, Harbecke *et al.* 2022). However, for *C. reinhardtii*, we also report Specificity (SP) and AUROC to enable direct comparison with existing predictors for this dataset. Detailed descriptions of all mentioned metrics are provided in Supplementary Section S10.

## 3 Results

In this section, we first present a cross-validation analysis of the training set of the *primary* dataset using various embeddings and wavelet functions. We then compare our proposed model, CaLMPhosKAN, against existing state-of-the-art methods on the *primary* independent test sets, as well as the other two additional datasets (*A549* and *C. reinhardtii*). Furthermore, we assess the predictive performance of CaLMPhosKAN on Intrinsically Disordered Regions (IDRs) and non-Intrinsically Disordered Regions (non-IDRs) of proteins. The results for IDR performance are provided in Supplementary Section S15, while the remaining findings are reported below.

### 3.1 Embeddings and wavlet transforms

We aim to assess the contribution of codon-aware embeddings to the final predictive performance of CaLMPhosKAN. To this end, we conducted 10-fold cross-validation (see Table 2) independently on CaLM embeddings (codon-aware), ProtTrans embeddings (amino acid-aware), and the fused representation (CaLM + ProtTrans). On the *primary S + T* set, CaLM embeddings produced a mean MCC, mean F1_wt_, and mean AUPR of 0.44±0.01, 0.71±0.01, and 0.80±0.01, respectively, which is lower than ProtTrans embeddings, which produced a mean MCC, mean F1_wt_, and mean AUPR of 0.46±0.01, 0.72±0.01, and 0.81±0.01, respectively. However, upon combining the two sets of embeddings via early fusion, as implemented in CaLMPhosKAN, we observed an improvement across all performance metrics, with a mean MCC, mean F1_wt_, and mean AUPR of 0.48±0.01, 0.74±0.01, and 0.83±0.01. Similarly, on the *primary Y* set, CaLM embeddings alone did not outperform ProtTrans embeddings. Yet, the combination of the two through early fusion resulted in better performance metrics than when either of the pLMs was used independently. These findings were further corroborated by evaluations on the *primary* independent *S + T* and *Y* test sets (see Supplementary Section S11), which confirmed the improvements observed during cross-validation. This improvement upon integration suggests that codon-aware embeddings contribute complementary information that enhances the overall model performance. Interestingly, on both the *primary S + T* and *Y* sets, CaLM did not independently surpass ProtTrans, which in fact deviates from the findings reported in the original CaLM paper (Outeiral and Deane 2024), where it significantly outperformed ProtTrans and other amino acid-aware embeddings in several protein-level tasks. Nonetheless, the performance differences were not substantial. This is particularly notable given that CaLM operates with roughly 33 times fewer parameters, highlighting its ability to deliver considerable predictive value with substantially reduced model complexity.

**Table 2.** Ten-fold cross-validation performance on *primary S + T* and *Y* sets.^a^

Set	Embeddings	MCC	PRE	REC	F1${}_{\text{wt}}$	AUPR
*S + T*	CaLM	0.44	0.74	0.68	0.71	0.80
	ProtTrans	0.46	0.75	0.69	0.72	0.81
	CaLMPhosKAN	**0.48**	**0.76**	**0.70**	**0.74**	**0.83**
*Y*	CaLM	0.32	0.67	0.60	0.65	0.69
	ProtTrans	0.33	0.67	0.62	0.66	0.70
	CaLMPhosKAN	**0.34**	**0.68**	**0.63**	**0.67**	**0.71**

a The highest values are bolded in each column. Note that the maximum standard deviation observed was 0.02.

**Figure 2.**
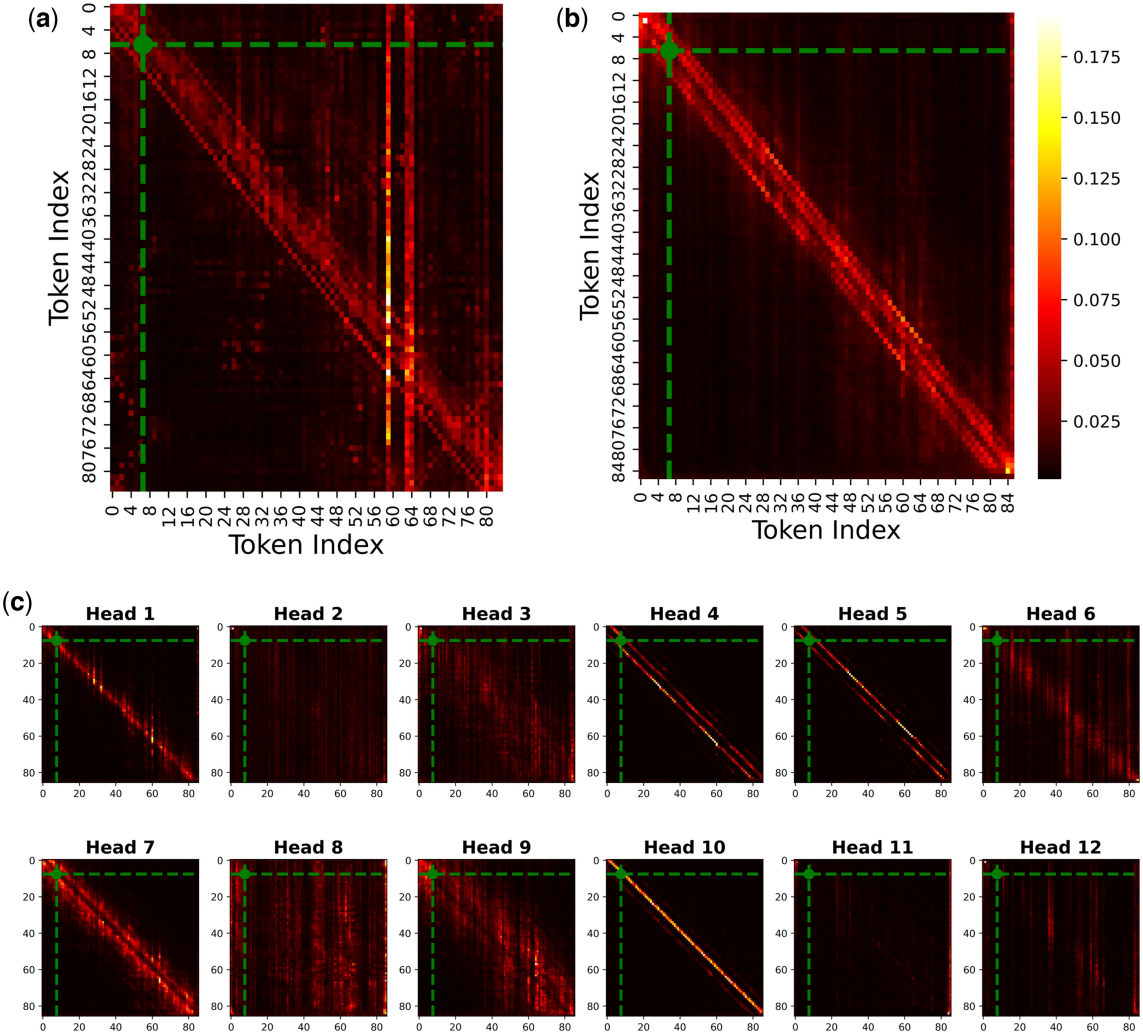
Heatmaps of averaged attention weights over the heads of the last encoder layer of (a) ProtTrans and (b) CaLM with protein P30047 as an input (86 tokens excluding <CLS> and <EOS>). The heatmaps in (c) display the individual attention heads from the last encoder layer of CaLM. The green dot in each denotes an experimentally annotated P-site (index: 7).

To better understand the observed improvement when integrating codon-aware and amino acid-aware embeddings, we analyzed the attention weights from both the CaLM and ProtTrans pLMs. Figure 2a and b illustrate heatmaps generated from the final layer of the encoder stacks in ProtTrans and CaLM respectively, averaged across all attention heads for a full input protein sequence (UniProt: P30047), consisting of 86 tokens excluding special tokens. Additionally, the head-wise heatmaps (32 per layer in ProtTrans and 12 per layer in CaLM) from the final encoder are also provided. The head-wise heatmaps for ProtTrans can be found in Supplementary Section S12, while those for CaLM are presented in Fig. 2c. It is important to note that, since the pLM encoders (CaLM and ProtTrans) are frozen during the training phase, the attention weights represent relationships learned during the pretraining process rather than patterns specific to phosphorylation sites prediction. As such, the attention maps reveal general token associations inherent to the pretraining data, which highlight the intrinsic relationships between residues. The heatmaps in Fig.2a and b illustrate this, showing a strong association between neighboring residues in both pLM encoders, thereby reinforcing the importance of window-level embeddings. Moreover, the heatmap from the ProtTrans encoder (see Fig. 2a) reveals strong associations with distant residues as well (for instance, token 7, denoted in green dot, shows high associativity with tokens 59 and 63), suggesting its ability to capture global sequence dependencies more effectively. In contrast, the attention distribution in the CaLM encoder is primarily skewed toward neighboring residues (see Fig. 2b), which might explain its relatively poorer performance when used independently. However, when examining the individual attention heads in the CaLM encoder (see Fig. 2c), some heads, such as head 6 and head 8, manage to capture associations with some distant residues, potentially adding useful information for prediction. Given that the attention heads across each pLM exhibit some varied patterns in their distribution of weights, the regions attended to by CaLM might differ from those attended to by ProtTrans, and this diversity likely contributes to the enhanced predictive performance observed when the embeddings from both models are combined.

**Figure 3.**
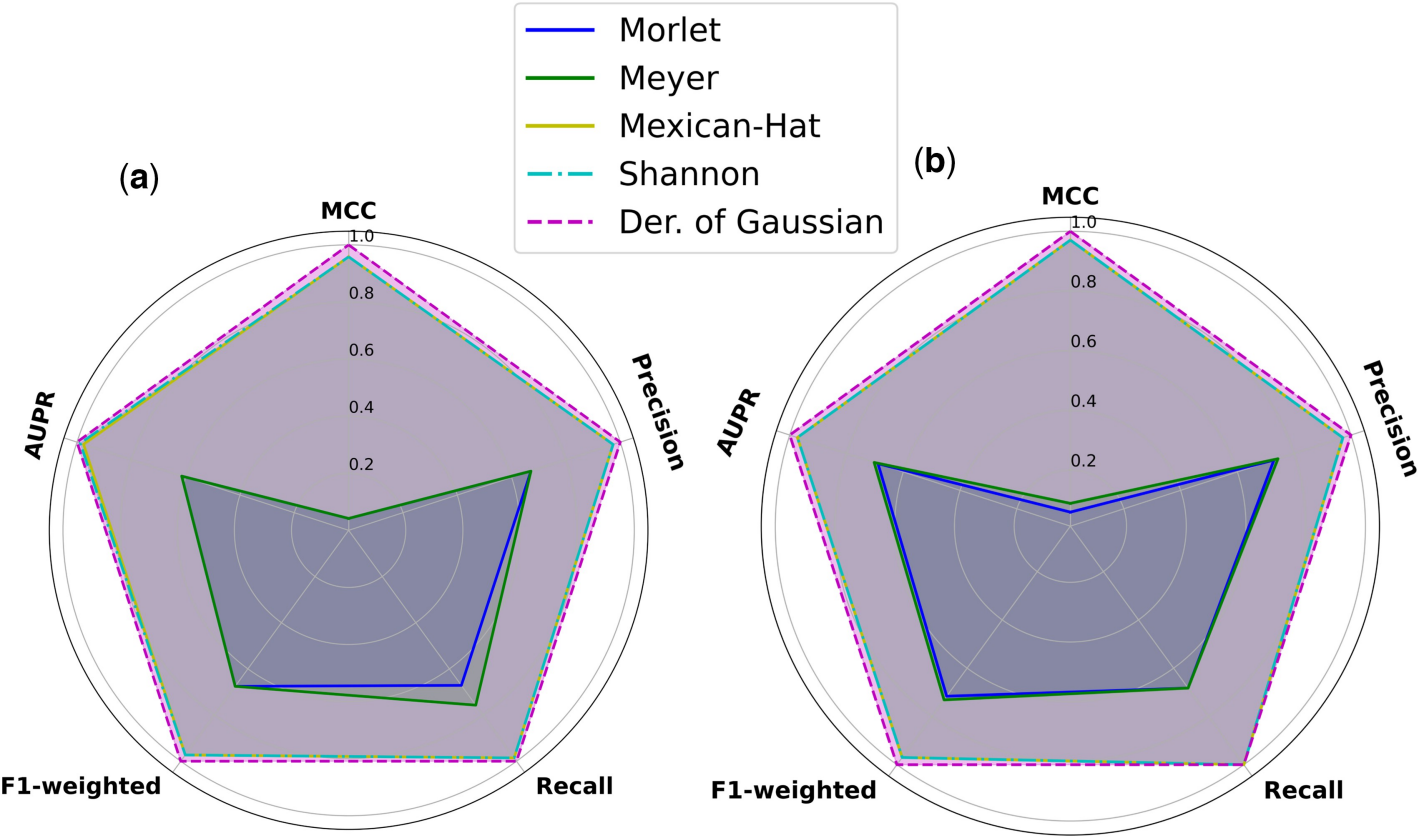
Radar plots comparing five wavelet functions across various performance metrics using 10-fold cross-validation. Each metric is normalized between 0 and 1 using max scaling (i.e. each value *x* in a feature column is divided by the maximum value of that column, $x^{\prime }=\frac{x}{\text{max}}$) to facilitate direct comparisons across wavelets. Plot (a) on the left shows results for the *primary S + T* set, and Plot (b) on the right shows results for the *primary Y* set.

Additionally, we examined the impact of varying the wavelet transform function used in the Wav-KAN module on the performance of CaLMPhosKAN. Using 10-fold cross-validation, we evaluated five distinct mother wavelet functions: Morlet, Meyer, Mexican Hat (or, Ricker), Shannon, and Derivative of Gaussian (DoG). Radar plots (with normalized metrics) for both the *primary S + T* and *Y* sets visually illustrate the comparative performance of each wavelet function (see Fig. 3). In these plots, the Morlet and Meyer wavelets showed limited coverage, indicating suboptimal performance. Conversely, the Mexican Hat and Shannon showed greater robustness in performance metrics with considerable coverage. In the *primary S + T* set, Shannon slightly led in AUPR, while in the *primary Y* set, its performance was on par with Mexican Hat (see Supplementary Section S13 for results in tabulated form). Most notably, the DoG wavelet covered the most expansive coverage on the plots (represented in purple), outperforming the others across all evaluated metrics. Consequently, the DoG wavelet was selected for integration into the Wav-KAN module of the CaLMPhosKAN.

### 3.2 Benchmarking with existing tools

The performance of CaLMPhosKAN was benchmarked on the *primary S + T* and *Y* independent test sets against five existing predictors: LMPhosSite (Pakhrin *et al.* 2023), DeepPSP (Guo *et al.* 2021), CapsNet (Wang *et al.* 2022), Musite (Wang *et al.* 2020), and MusiteDeep (Wang *et al.* 2020). Notably, LMPhosSite and DeepPSP are the most recent predictors. For DeepPSP, prediction results were extracted from their GitHub repository, and performance was computed on the subset of samples corresponding to our test sets (*aka primary* test sets). Meanwhile, LMPhosSite was re-implemented by training and testing on our *primary* train and test datasets, respectively. For the remaining predictors, performance metrics were directly adopted from DeepPSP’s literature (Guo *et al.* 2021), as our test set is a subset of their test set with a similar number of sites. Table 3 summarizes the comparative performance of CaLMPhosKAN and the five existing predictors based on six measures: MCC, PRE, REC, F1, F1_wt_, and AUPR (see Supplementary Section S14 for PR curves). On the *primary S + T* set, CaLMPhosKAN achieved an MCC of 0.41, F1_wt_ of 0.51, and an AUPR of 0.53, demonstrating improvements across these three key metrics over the current best performing predictors, LMPhosSite and DeepPSP. A similar trend was observed on the *primary Y* set, where CaLMPhosKAN achieved an MCC of 0.30, F1_wt_ of 0.78, and an AUPR of 0.42, surpassing LMPhosSite and DeepPSP on these metrics.

**Table 3.** Performance comparison on *primary* independent test sets and *A549* test set.^a^

Set	Predictor	MCC	PRE	REC	F1	F1_wt_	AUPR
*S + T* (*Primary*)	CapsNet	0.27	0.24	**0.88**	0.38	N/A	0.31
	Musite	0.20	0.22	0.76	0.35	N/A	0.33
	MusiteDeep	0.33	0.32	0.70	0.44	N/A	0.46
	DeepPSP	0.38	0.39	0.69	0.48	0.79	0.51
	LMPhosSite	0.39	0.35	0.79	0.49	0.75	0.31
	CaLMPhosKAN	**0.41**	**0.47**	0.57	**0.51**	**0.83**	**0.53**
*Y* (*Primary*)	CapsNet	0.20	0.23	**0.88**	0.37	N/A	0.19
	Musite	0.14	0.24	0.68	0.35	N/A	0.28
	MusiteDeep	0.20	0.35	0.35	0.35	N/A	0.33
	DeepPSP	0.26	0.32	0.66	0.42	0.70	0.39
	LMPhosSite	0.28	0.33	0.63	**0.44**	0.72	0.28
	CaLMPhosKAN	**0.30**	**0.41**	0.46	**0.44**	**0.78**	**0.42**
*S + T* (*A549*)	DeepPSP	0.45	0.54	**0.85**	0.66	0.69	0.65
	LMPhosSite	0.47	**0.83**	0.58	0.68	0.71	0.70
	CaLMPhosKAN	**0.48**	0.73	0.76	**0.75**	**0.73**	**0.79**

a The highest values are bolded in each column.

Subsequently, we evaluated the generalizability of CaLMPhosKAN using the *A549* and *C. reinhardtii* datasets. On the *A549 S + T* test set, we benchmarked the CaLMPhosKAN model, trained on the *primary S + T* train set, against LMPhosSite and DeepPSP (refer to last three rows of Table 3). CaLMPhosKAN demonstrated superior performance, achieving the highest MCC, F1_wt_, and AUPR values among the compared models. Next, we evaluated CaLMPhosKAN on the *C. reinhardtii* dataset, which includes *S + T* training and independent test sets. Here, CaLMPhosKAN was trained on the *C. reinhardtii S + T* training set and tested on its corresponding test set. Performance metrics for existing *C. reinhardtii*-specific predictors (DeepPhos, Chlamy-MwPhosSite, and Chlamy-EnPhosSite) were obtained from the same source literature (Thapa *et al.* 2021), from which our dataset was derived. Note that these metrics are different from the previous results tables (i.e. Tables 2 and 3) to facilitate a fair comparison with existing predictors. As reported in Table 4, CaLMPhosKAN outperformed these predictors, underlining its adaptability and effectiveness in phosphorylation sites prediction across organisms beyond humans. It is also worth noting that for balanced or nearly balanced test sets (*A549* and *C. reinhardtii*), CaLMPhosKAN exhibited the best balance between PRE and REC in *A549* and between SP and SN in *C. reinhardtii*.

**Table 4.** Performance comparison on *C. reinhardtii S + T* independent test set.^a^

Predictor	MCC	SP	SN (REC)	AUROC
DeepPhos	0.61	0.77	0.83	0.88
Chlamy-MwPhosSite	0.64	0.78	0.86	0.90
Chlamy-EnPhosSite	0.64	0.73	**0.90**	0.90
CaLMPhosKAN	**0.67**	**0.84**	0.83	**0.92**

a The highest values are bolded in each column.

## 4 Conclusion

The codon language model has shown impressive performance in various protein-level tasks (Outeiral and Deane 2024). In this work, we applied it to phosphorylation sites prediction, a well-studied residue-level task. We developed a framework that translates amino acid sequences into reliable coding sequences using a dynamic programming-based procedure and acquired codon-aware embeddings via the codon language model (i.e. CaLM). The target sites were represented at the window levels, preserving global context to capture both proximal residue associations and full sequence dependencies. Our analysis revealed that CaLMPhosKAN effectively captures information complementary to amino acid-level embeddings by incorporating codon-level embeddings. This bimodal representation improved predictive performance and highlighted potential roles for codon usage in phosphorylation, such as its influence on translational efficiency and kinase accessibility. Attention weight analysis further indicated associations between residues and their neighbors, offering insights into local interactions. Moreover, the improved performance in intrinsically disordered regions further suggests that codon-level information aids in modeling flexibility and disorder, contributing to the understanding of phosphorylation regulation in signaling and regulatory functions.

Our framework, CaLMPhosKAN, outperformed existing approaches across multiple datasets. This approach of integrating codon-level information can be extended to other residue-level prediction tasks, with potential enhancements through the incorporation of additional modalities such as structure-aware embeddings. In future work, we plan to explore and validate the insights gained from this study further by using experimentally derived datasets, kinase-specific substrates, and motif enrichment analysis to bridge computational predictions with biological interpretation.

## Supplementary Material

btaf124_Supplementary_Data

## Data Availability

The underlying data are available at https://github.com/KCLabMTU/CaLMPhosKAN.
