# Supplementary material for: CaLMPhosKAN: prediction of general phosphorylation sites in proteins via fusion of codon aware embeddings with amino acid aware embeddings and wavelet-based Kolmogorov–Arnold network
Source: Bioinformatics. 2025 Mar 21;41(4):btaf124. doi: 10.1093/bioinformatics/btaf124 (PMC11972116; doi:10.1093/bioinformatics/btaf124)
Supplement: btaf124_Supplementary_Data [file btaf124_supplementary_data.pdf]

CaLMPhosKAN: Prediction of General Phosphorylation Sites in Proteins via Fusion of Codon Aware Embeddings with Amino Acid Aware Embeddings and Wavelet-based Kolmogorov–Arnold Network

Pawel Pratyush [1], Callen Carrier[2], Suresh Pokharel [1], Hamid D. Ismail [3], Meenal Chaudhari [4], Dukka B. KC [1,2,*]

[1]Golisano College of Computing and Information Sciences, Rochester Institute of Technology, Rochester NY 14623, USA.

[2]College of Computing, Michigan Technological University, Houghton MI 49931, USA.

[3]College of Engineering, North Carolina A&T State University, Greensboro NC 27411, USA.

[4]College of Applied Sciences and Technology, Illinois State University, Normal IL 61761, USA.

*correspondence to dkcvcs@rit.edu

Supplementary Sections

Table of Contents | Page

I. **Section S1.** Description of existing general phosphorylation site predictors......2

II. **Section S2.** Ablation study on CD-Hit thresholds/cut-offs......3

III. **Section S3**. Curation steps of *A549* and *Chlamydomonas reinhardtii* datasets 3

IV. **Section S4**. Change in the count of sites before reverse translation vs after translation of DeepPSP dataset.....4

V. **Section S5**. Recent Phosphorylation Site Predictors with Residue-Specific Models......5

VI. **Section S6**. Ablation study on different embeddings and network types......6

VII. **Section S7**. Comparison between MLP and KAN......6

VIII. **Section S8**. Details of architectures of ConvBiGRU with Wav-KAN......7

IX. **Section S9.** Learning Curves......8

X. **Section S10**. Performance Measures......9

XI. **Section S11.** CaLMPhosKAN Performance on *primary S+T* and *Y* independent test sets......10

XII. **Section S12.** Individual Attention Heads Heatmaps......10

XIII. **Section S13.** 10-fold cross-validation on different wavelet transforms....11

XIV. **Section S14.** Precision-Recall (PR) Curves....12

XV. **Section S15.** Evaluation of CaLMPhosKAN on Intrinsically Disordered Regions 12

XVI. **References** 15

Section S1 Description of existing phosphorylation site predictors

Predictor	Encoding	Input Sequence type	Limitation
**RFPhos [1]**	Uses physicochemical properties derived from the peptide sequence	Peptide	• Relies on handcrafted features<br>• Relies on isolated window fragments.<br>• Limited global context
**GPS [2]**	Physicochemical properties derived from the peptide sequence	Peptide	• Relies on handcrafted features<br>• Relies on isolated window fragments.<br>• Limited global context
**Musite [3]**	KNN features and Physicochemical properties derived from the peptide sequence	Peptide	• Relies on handcrafted features<br>• Relies on isolated window fragments.<br>• Limited global context
**MusiteDeep [4]**	One-hot encoding of peptide sequences	Peptide	• Relies on isolated window fragments.<br>• Limited global context
**CapsNet [5]**	Physicochemical properties derived from the peptide sequence	Peptide	• Relies on handcrafted features<br>• Relies on isolated window fragments.<br>• Limited global context
**DeepPhos[6]**	One hot encoding with three different window lengths	Peptide	• Relies on isolated window fragments<br>• Limited global context
**Transphos [7]**	Encodes peptide sequence of three different lengths using a word embedding followed by transformer encoders	Peptide	• Relies on isolated window fragments.<br>• Limited global context
**Attenphos [8]**	Encodes peptide sequence of three different lengths using a word embedding followed by sef-attention heads	Peptide	• Relies on isolated window fragments.<br>• Limited global context
**Chlamy-EnPhosSite [14]**	Encodes multi-window peptide sequences through supervised word embedding layer	Peptide	• Relies on isolated window fragments.<br>• Limited global context<br>• Supervised embedding could cause target information leakage to the feature set.

**DeepPSP [9]**	Uses one-hot encoding to embed peptides for local and 2000-length sequences for global.	Peptide and 2000-length sequence	• Relies on isolated window fragments (local module).<br>• The global module has a length limitation.<br>• The global module has a minimal contribution to the final performance
**LMPhosSite [10]**	Combination of pLM embedding (generated from the full sequence) and supervised word embedding (generated from the peptide sequence).	Full sequence and peptide	• Uses per-residue embedding from pLM confined to the target site only.<br>• Also relies on isolated fragment sequences (for the supervised embedding layer).<br>• Supervised embedding could cause target information leakage to the feature set.

Section S2 Ablation study on CD-HIT thresholds/cut-offs

**CD-HIT cut-off**	**#proteins**	**Set**	**#+ve/-ve sites**	**Model**	**Independent Testing**		
					**MCC**	**$F1_{wt}$**	**AUPR**
0.3	9139	Train<br>Test	129,170/647,809<br>16,964/85,057	CaLM	0.36	0.78	0.50
				ProtTrans	0.38	0.80	0.51
				CaLMPhosKAN	0.40	0.81	0.52
0.4	10138	Train<br>Test	141,372/720,976<br>16,964/85,057	CaLM	0.38	0.79	0.51
				ProtTrans	0.39	0.82	0.52
				CaLMPhosKAN	0.41	0.83	0.52
0.5 (ours)	11365	Train<br>Test	154,220/800,329<br>16,964/85,057	CaLM	0.37	0.79	0.50
				ProtTrans	0.40	0.82	0.52
				CaLMPhosKAN	0.41	0.83	0.53

Section S3 Curation steps of *A549* and *Chlamydomonas reinhardtii* datasets

***A549* test set:**

The dataset was sourced from Lv *et al.* [15], consisting of experimentally validated phosphorylation sites of human *A549* cells infected with SARS-CoV-2. Initially, it consisted of 14,119 positive sites, which were processed through homology removal using CD-HIT with a cutoff of 0.30. Unannotated sites within the same sequences were considered negative sites, and the dataset was balanced using random undersampling of the negative set. It was then split into a training and independent test set at an 80:20 ratio, resulting in 4308 *S+T*

sites each for the positive and negative sets in the training set and 1079 *S+T* sites each for the positive and negative sets in the independent test set. However, the number of *S+T* sites in the training set was relatively limited, restricting the robustness of model training. To address this limitation, we combined the training and independent test sets into a single set and subsequently filtered this set to remove any overlap with our *primary* *S+T* training set used in this study. This resulted in a final set consisting of 1144 positive sites and 1049 negative sites confined to *S+T* residues. We refer to this curated set as the *A549* test set, which was treated as an additional independent test set alongside the *primary* independent test set already in use. The inclusion of this additional test set allowed for a more extended evaluation of the generalizability of our CaLMPhosKAN model.

Chlamydomonas reinhardtii (C. reinhardtii) **training and test set:**

The phosphorylation dataset for the *Chlamydomonas reinhardtii* organism was obtained from Thapa *et al.*[14], who originally sourced their data from Wang et al. [16]. All phosphorylation sites were experimentally detected and cross-referenced with the Joint Genome Institute's C. reinhardtii database v5.6, supplemented with the NCBI chloroplast (BK000554) and mitochondrial (NC_001638.1) databases to ensure complete protein sequence coverage. All S, T, and Y residues not identified as phosphorylation sites were considered part of the negative dataset. Due to the limited number of phosphorylated Y sites, these were excluded from further analysis. The S and T sites were combined into a single S+T dataset for model development. The dataset was then split into an 80:20 ratio for training and independent testing. The training set contained 17,345 phosphorylated *S+T* sites as the positive set and 460,015 sites as the negative set. The independent test set consisted of 4,338 positive and 115,005 negative *S+T* sites. Following the preprocessing steps of Thapa *et al.*[14], both the training and independent test sets were balanced through random undersampling.

<u>*Note for reverse translation of C. reinhardtii dataset*</u>: Since UniProt IDs were not originally present in this dataset, reverse translation posed a challenge, as the procedure described in *Section 2.1.2* of the manuscript requires mapping UniProt IDs to RefSeq IDs. To overcome this, the transcription sequences from different versions of the Phytozome database [17] were downloaded and combined into a single FASTA file as a database of sequences. The defline of each sequence in this database includes the gene name and transcript ID. An exhaustive search was conducted using the *C. reinhardtii* training and test datasets against the pooled transcription sequences in the database. The ID numbers and protein sequences in these datasets were matched against the ID numbers and transcript sequences in the FASTA file, allowing us to successfully obtain the corresponding UniProt ID for each sequence.

Section S4 Change in the count of sites before vs after reverse translation of DeepPSP dataset

As *Section 2.1.2* of the manuscript mentioned, proteins with less than 100% alignment identity were excluded during the protein-coding DNA translation (aka 'reverse translation') process. Additionally, proteins without corresponding RefSeq identifiers were discarded. Also, some sites that no longer matched the original annotation due to updates in UniProt were also removed. In Table S4(a), we present the original dataset curated by Li *et* al. [9] for their DeepPSP predictor. In Table S4(b), we show the dataset obtained after our translation process, which was used to build the CaLMPhosKAN predictor. We refer to this reduced dataset throughout as the *primary* dataset. To ensure a fair comparison, we used the DeepPSP model to predict the sites corresponding to our test set (*primary* test set), as shown in Table S4(b).

Table S4: Number of positive and negative sites in train and test set **(a)** before reverse translation (DeepPSP dataset) and **(b)** after translation.

(a) before translation (original dataset)

Set	Target Residue	#P-sites (+ve)	#NP-sites (-ve)
Train	*S+T*	165,787	879,507
	Y	28,965	134,997
Test	*S+T*	18,588	102,113
	Y	3248	14,504

(b) after translation (our *primary* dataset)

Set	Target Residue	#P-sites (+ve)	#NP-sites (-ve)
Train	*S+T*	154,220	800,329
	Y	27,077	123,918
Test	*S+T*	16,964	84,057
	Y	3054	13,347

Section S5 Recent Phosphorylation Site Predictors with Residue-Specific Models

Table S5 presents examples of recently developed phosphosite predictors that utilize residue-specific models. These models either combine S and T while treating Y separately or develop distinct models for each residue type (S, T, and Y).

Table S5: Some prominent examples of recently developed phosphosite predictors that utilize residue-specific models

Predictor	Year	Description
**MusiteDeep**	2020	One model for S/T and another model for Y
**DeepIPS**	2021	One model for S/T and another model for Y
**Chlamy-EnPhosSite**	2021	Separate models for S, T, and Y, and as well as combined ST model
**MeL-STPhos**	2024	One model for S/T and another model for Y
**TransPhos**	2022	Separate Models for S, T, and Y
**Attentionphos**	2024	Separate Models for S, T, and Y
**GPS 6.0**	2023	One model for S/T and another model for Y
**KinasePhos 3.0**	2023	One model for S/T and another model for Y
**LMPhosSite**	2023	One model for S/T and another model for Y

Section S6 Ablation study on different embeddings and network types

We conducted an ablation study on different network architectures and embedding types, evaluating CaLM, ESM-2, and ProtTrans (ProtT5 in this study) embeddings using three different architectures with 10-fold cross-validation on the *primary S+T* dataset. Across all embedding types, ConvBiGRU consistently achieved the best MCC and AUPR scores. Additionally, we observed that ESM-2 performed on par with ProtT5, highlighting its potential as a strong alternative (see Table S6(a)).

Table S6(a): Ablation on individual embeddings (CaLM, ESM-2 and ProtTrans)

Embedding	Model	MCC	PRE	REC	$F1_{wt}$	AUPR
**CaLM**	CNN	0.41±0.01	0.70±0.01	0.67±0.01	0.69±0.01	0.78±0.01
	ConvGRU	0.43±0.01	0.73±0.02	0.68±0.02	0.70±0.01	0.79±0.01
	ConvBiGRU	0.44±0.01	0.74±0.02	0.68±0.02	0.71±0.01	0.80±0.01
**ESM-2**	CNN	0.45 ± 0.01	0.73 ±0.02	0.74 ±0.02	0.73 ±0.01	0.81 ± 0.01
	ConvGRU	0.45 ± 0.01	0.74 ±0.02	0.68 ±0.03	0.71 ±0.01	0.81 ± 0.01
	ConvBiGRU	0.46± 0.01	0.74 ±0.02	0.70 ±0.02	0.72 ±0.01	0.81 ± 0.01
**ProtTrans**	CNN	0.45 ± 0.01	0.74 ±0.02	0.68 ±0.02	0.71 ±0.01	0.80 ± 0.01
	ConvGRU	0.45 ± 0.01	0.73±0.01	0.70 ±0.02	0.71 ±0.01	0.81 ± 0.01
	ConvBiGRU	0.46±0.01	0.75±0.01	0.69±0.02	0.72±0.01	0.81±0.01

Furthermore, we performed an ablation study on fused embeddings used in the *Embedding Extraction and Fusion (EEF) Module* of our architecture. The observed performance trends in individual embeddings remained consistent when fused embeddings were used (see Table S6(b)), reinforcing our initial design choices.

Table S6(b): Ablation on fused embeddings

Embedding	Model	MCC	PRE	REC	$F1_{wt}$	AUPR
**Fused**	CNN	0.45±0.01	0.74 ±0.01	0.70±0.01	0.72±0.01	0.81 ± 0.01
	ConvGRU	0.46±0.01	0.76 ±0.01	0.69±0.01	0.73±0.01	0.82 ± 0.01
	ConvBiGRU-MLP	0.47±0.01	0.76 ±0.01	0.70±0.01	0.74±0.01	0.83 ± 0.01
	ConvBiGRU-KAN	0.48±0.01	0.76±0.01	0.70±0.01	0.74±0.01	0.83±0.01

Section S7 Comparison between MLP and KAN

Table S7: Comparision of KAN (CaLMPhosKAN) against MLP (CaLMPhosMLP) across *primary S+T* and *Y* datasets

Dataset	Embeddings	MCC	PRE	REC	$F1_{wt}$	AUPR
S+T	CaLMPhosKAN	0.48±0.01	0.76±0.01	0.70±0.01	0.74±0.01	0.83±0.01
	CaLMPhosMLP	0.47±0.01	0.76 ± 0.01	0.70±0.01	0.74±0.01	0.83 ± 0.01
Y	CaLMPhosKAN	0.34±0.01	0.68±0.01	0.63±0.02	0.67±0.01	0.71±0.01
	CaLMPhosMLP	0.33 ± 0.01	0.68 ± 0.01	0.62± 0.02	0.66 ± 0.01	0.71 ± 0.01

Section S8 Details of architectures of ConvBiGRU with Wav-KAN

1) *S+T* Model

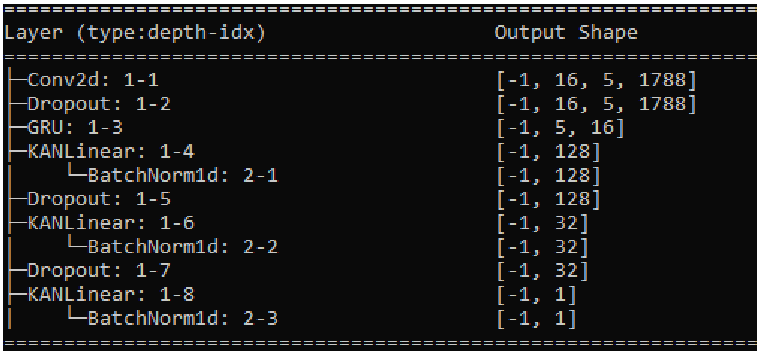

```
==============================================================
Layer (type:depth-idx)                   Output Shape
==============================================================
├─Conv2d: 1-1                            [-1, 16, 5, 1788]
├─Dropout: 1-2                           [-1, 16, 5, 1788]
├─GRU: 1-3                               [-1, 5, 16]
├─KANLinear: 1-4                         [-1, 128]
│    └─BatchNorm1d: 2-1                  [-1, 128]
├─Dropout: 1-5                           [-1, 128]
├─KANLinear: 1-6                         [-1, 32]
│    └─BatchNorm1d: 2-2                  [-1, 32]
├─Dropout: 1-7                           [-1, 32]
├─KANLinear: 1-8                         [-1, 1]
│    └─BatchNorm1d: 2-3                  [-1, 1]
==============================================================
```

Figure S8.1: *S+T* Model configurations and the output shape of each layer

- **Input Layer:** The model takes an input with a shape of (batch_size, 1, window_size, feature_dimension). Here, 1 indicates the number of input channels, window_size is 9 and feature dimension is 1792.
- **First Conv2D Layer**: This layer applies a 2D convolution with 16 filters of size 5x5 across the input. There is no padding.
- **Dropout Layer:** Directly following the convolutional operation with a rate of 0.3.
- **Dimension Adjustment for GRU Input:** Post-convolution and before feeding into the GRU:
  Convolutional Output Height Calculation: Derived from the window size minus four, reflecting the reduction from the convolution operation without padding.
  Convolutional Output Width Calculation: Similar reduction for the feature dimension (feature dimension minus four).
  These dimensions determine the number of features per timestep that will be fed into the GRU.
  Permutation and Reshaping for GRU: The output of the convolutional layer is permuted and reshaped to align with the requirements of the GRU layer:
  - The output tensor is permuted to place the feature dimension (channels from the Conv2D layer) contiguous to the sequence dimension, facilitating the reshaping operation.
  - It's then reshaped to merge the height and remaining width, forming the input sequence for the GRU where each sequence step contains the flattened features of the respective window slice.
- **BiGRU Layer**: A bidirectional GRU processes the reshaped input from Conv2D. Contains 8 units.
- **Fully Connected KAN Layers:**

First Wav-KAN Linear Layer: A Kolmogorov-Arnold Network (KAN) applies transformations aided by the DoG wavelet transform. Contains 128 neurons.
Dropout Layer: A dropout rate of 0.3 is applied.
Second Wav-KAN Linear Layer: A DoG wavelet KAN layer with 32 neurons.
Dropout Layer: A dropout rate of 0.3 is applied
(Note: Batch Normalization is used before each Linear layer)
Output Layer: contains a single neuron.

2) *Y* Model

```
===============================================================
Layer (type:depth-idx)                   Output Shape
===============================================================
├─Conv2d: 1-1                            [-1, 16, 5, 1788]
├─Dropout: 1-2                           [-1, 16, 5, 1788]
├─GRU: 1-3                               [-1, 5, 16]
├─KANLinear: 1-4                         [-1, 24]
│    └─BatchNorm1d: 2-1                  [-1, 24]
├─Dropout: 1-5                           [-1, 24]
├─KANLinear: 1-6                         [-1, 1]
│    └─BatchNorm1d: 2-2                  [-1, 1]
===============================================================
```

Figure S8.2: *Y* Model configurations and the output shape of each layer

- **Input Layer:** The model takes an input with a shape of (batch_size, 1, window_size, feature_dimension). Here, 1 indicates the number of input channels, window_size is 9 and feature dimension is 1792.
- **First Conv2D Layer**: Applies a 2D convolution with 16 filters of size 5x5 across the input. No padding.
- **Dropout Layer:** Rate of 0.3.
- **Dimension Adjustment for GRU Input:** Post-convolution and before feeding into the GRU. (Identical to *the S+T* model).
- **BiGRU Layer**: A bidirectional GRU processes the reshaped input from Conv2D. Contains 8 units.
- **Fully Connected KAN Layers:**
  Single Wav-KAN Linear Layer: Contains 24 neurons with DoG wavelet.
  Dropout Layer: A dropout rate of 0.3 is applied
  (Note: Batch Normalization is used before the Linear layer)
  Output Layer: contains a single neuron.

Section S9 Learning Curves

Below are some sample loss/accuracy curves obtained during the cross-validation of CaLMPhosKAN on the *S+T* dataset. Note that early stopping was used in these experiments, and slight overfitting can be observed towards the end.

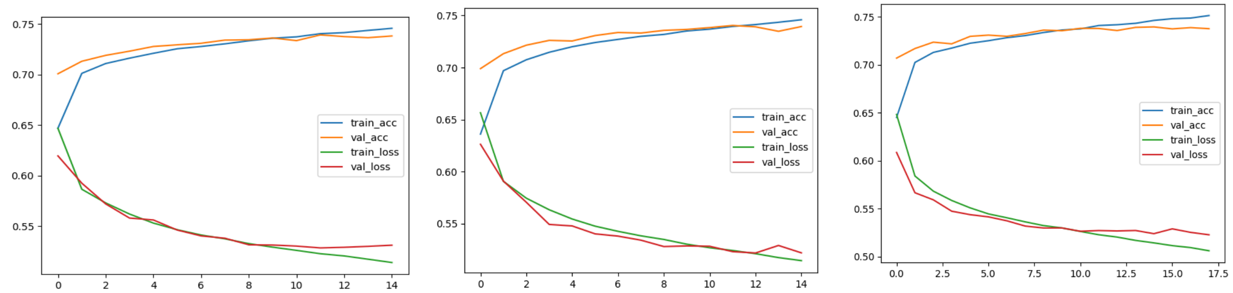

Figure S9: Learning Curves Samples from Cross-validation of CaLMPhosKAN

Section S10 Performance Measures

Let **TP** (True Positive) be the count of the predicted P-sites (phosphorylated sites), **TN** (True Negative) be the count of correctly predicted NP-sites (non-phosphorylated sites), **FP** (False Positive) be the count of incorrectly predicted P-sites, and **FN** (False Negative) be the count of incorrectly predicted NP-sites. Based on these fundamental metrics we define the following measures:

- **Matthews Correlation Coefficient (MCC)** = $\frac{(TP \times TN) - (FP \times FN)}{\sqrt[2]{(TP + FP)(TP + FN)(TN + FP)(TP + FN)}}$ (range $\in$ [-1,1])
- **F1** = $\frac{TP}{TP+0.5(FN+FN)}$ (range $\in$ [0,1])
- **F1$_{\text{weighted}}$ (F1$_{\text{wt}}$)** = $\sum_{i=1}^{n} (\frac{S_i}{S} \times F1_i)$ (range $\in$ [0,1])

  Where,
  - n is the number of classes (=2, in this case).
  - S_i is the support (number of true instances) for class i.
  - S is the total number of instances across all classes.
  - $F1_i$ is the F1 score for the ith class.
- **Precision (PRE)** = $\frac{TP}{TP+FP}$ (range $\in$ [0,1])
- **Recall (REC) (or, Sensitivity/SN)** = $\frac{TP}{TP+FN}$ (range $\in$ [0,1])
- **Area Under Precision-Recall Curve (AUPR/PrAUC):** Area under the curve of precision plotted against recall (Sensitivity) at various decision thresholds with cut-offs ranging from 0.0 to 1.0. The range of AUROC is [0,1].

You may notice that for the *C. reinhardtii* dataset testing, we incorporated additional evaluation metrics, including Specificity (SP) and AUROC, to ensure a fair comparison with existing predictors. These metrics are defined below:

- **Specificity (SP)** = $\frac{TN}{FP+TN}$ (range $\in$ [0,1])
- ***Area* Under Receiver Operating Characteristic Curve (AUROC)**: Area under the curve of Sensitivity plotted against (1- Specificity) at various decision threshold cut-offs ranging from 0.0 to 1.0. The range of AUROC is [0,1].

Section S11 CaLMPhosKAN performance on the *primary* *S*+*T* and *Y* independent test sets

Table S11: performance of different embedding combinations (CaLM, ProtTrans, and CaLM+ProtTrans (i.e., CaLMPhosKAN)) on the *primary* *S*+*T* and *Y* independent test sets.

Embeddings	Serine + Threonine (*S*+*T*)			Tyrosine (*Y*)		
	**MCC**	**$F1_{wt}$**	**AUPR**	**MCC**	**$F1_{wt}$**	**AUPR**
CaLM	0.37	0.79	0.50	0.27	0.75	0.41
ProtTrans	0.40	0.82	0.52	0.29	0.77	0.42
CaLMPhosKAN	0.41	0.83	0.53	0.30	0.78	0.42

Section S12 Individual Attention Heads Heatmaps

The heatmaps for individual heads from the final encoder layer of the ProtTrans (specifically, ProtT5) encoder are displayed here. These visualizations are generated for a sample protein sequence with a length of 86. The ProtTrans encoder features 32 heads per layer.

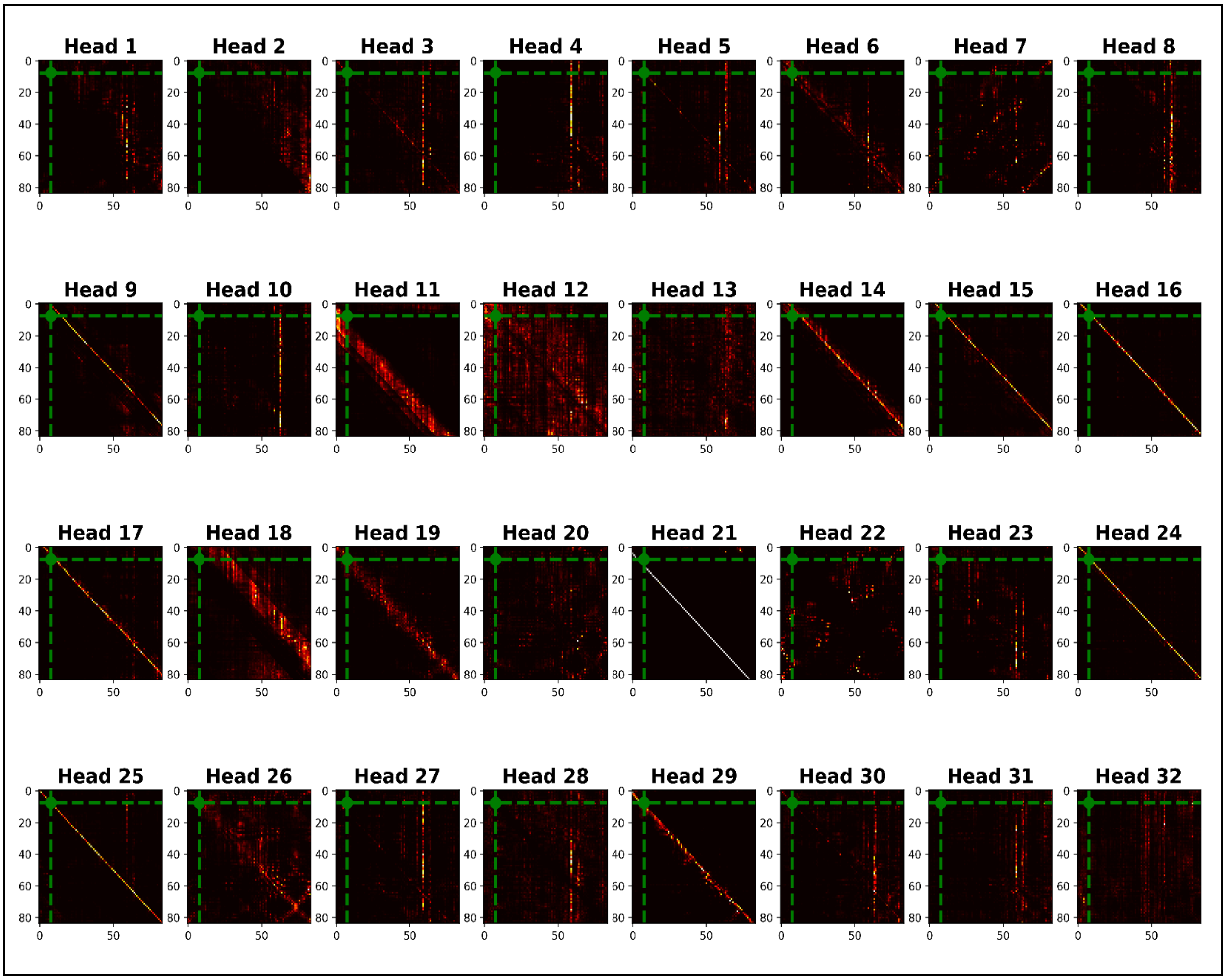

Figure S12: ProtTrans averaged attention map from the final encoder layer

Section S13 10-fold cross-validation on different wavelet transforms

Table S13 presents 10-fold cross-validation mean MCC, mean PRE, mean REC, mean $F1_{wt}$, and mean AUPR of five mother wavelets explored in this work.

Table S13: Comparison of 10-fold cross-validation for five mother wavelets

Dataset	Wavelet	MCC	PRE	REC	$F1_{wt}$	AUPR
Serine + Threonine (S+T)	Morelet	0.02	0.51	0.47	0.50	0.51
	Meyer	0.02	0.51	0.53	0.50	0.51
	Mexican-Hat	0.46	0.74	0.69	0.72	0.81
	Shannon	0.46	0.74	0.69	0.72	0.82
	Der. of Gaussian (DoG)	0.48	0.76	0.70	0.74	0.83
Tyrosine	Morelet	0.02	0.51	0.43	0.48	0.51
	Meyer	0.03	0.52	0.43	0.49	0.52

(Y)	Mexican-Hat	0.33	0.68	0.63	0.65	0.72
	Shannon	0.33	0.68	0.63	0.65	0.72
	Der. of Gaussian (DoG)	0.34	0.70	0.63	0.67	0.74

Section S14 Precision-Recall (PR) Curves

The PR curves for the S+T model and Y model on the *primary* independent test set are presented in Figure S14. It is important to note that this test set is highly balanced. CaLMPhosKAN demonstrates a higher overall AUPR in both the S+T and Y models. Additionally, CaLMPhosKAN exhibits higher precision in the lower recall range (0.0 to 0.6). This is particularly crucial in imbalanced datasets, where positive samples are scarce, and performance in the lower recall range becomes critical. Superior precision at lower recall indicates that CaLMPhosKAN handles the minority class (i.e. positive class) more effectively, making it better suited to address the inherent challenges posed by class imbalance in real-world scenarios. In contrast, DeepPSP performs better in the higher recall range (0.6 to 1.0), suggesting that DeepPSP might make more positive predictions but with lower confidence, potentially increasing false positives.

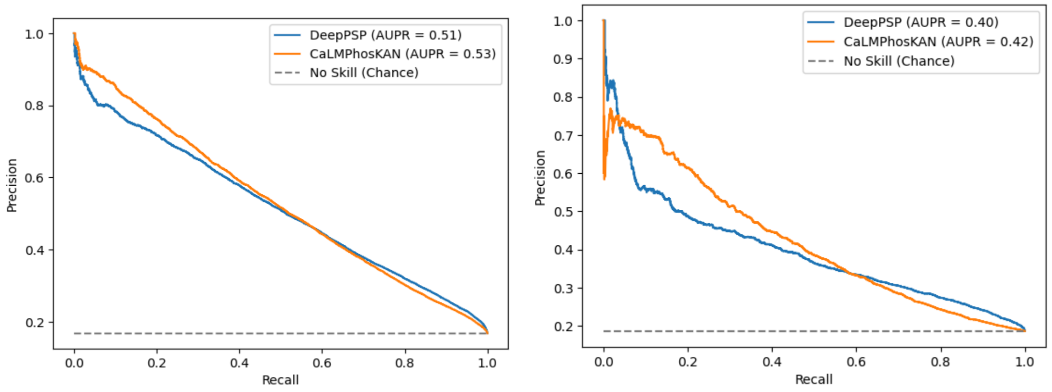

(a) **(b)**

Figure S14. PR curves for **(a)** the S+T Model and **(b)** the Y Model on the *primary* independent test set.

Section S15 Evaluation of CaLMPhosKAN on Intrinsically Disordered Regions

IDRs and non-IDRs exhibit distinct structural and functional properties that can influence phosphorylation. Specifically, IDRs are often enriched in P-sites because their ability to adopt multiple conformations makes them accessible to kinases [12]. Here, we assess the predictive performance of CaLMPhosKAN specifically in regions of disorder and order. We utilized the popular protein intrinsic disorder region (IDR) prediction tool, flDPnn [13], to identify S and T sites of the *primary* dataset located within disordered and non-disordered regions on proteins in the test set. Subsequently, we segregated the sites based on their regions, disordered and non-disordered, and applied our tool, CaLMPhosKAN, to each set separately. For benchmarking, we compared our results with the current predictor, DeepPSP, by evaluating its performance on both the disordered and non-disordered datasets. From the bar graphs in Figure S15.1, it can be observed that CaLMPhosKAN achieves better MCC, $F1_{wt}$, and AUPR than DeepPSP in both IDR and non-IDR regions. Notably, there appears to be a difference in the performance of these models in inter-regions, which can be attributed to the variation in the distribution of P and NP sites.

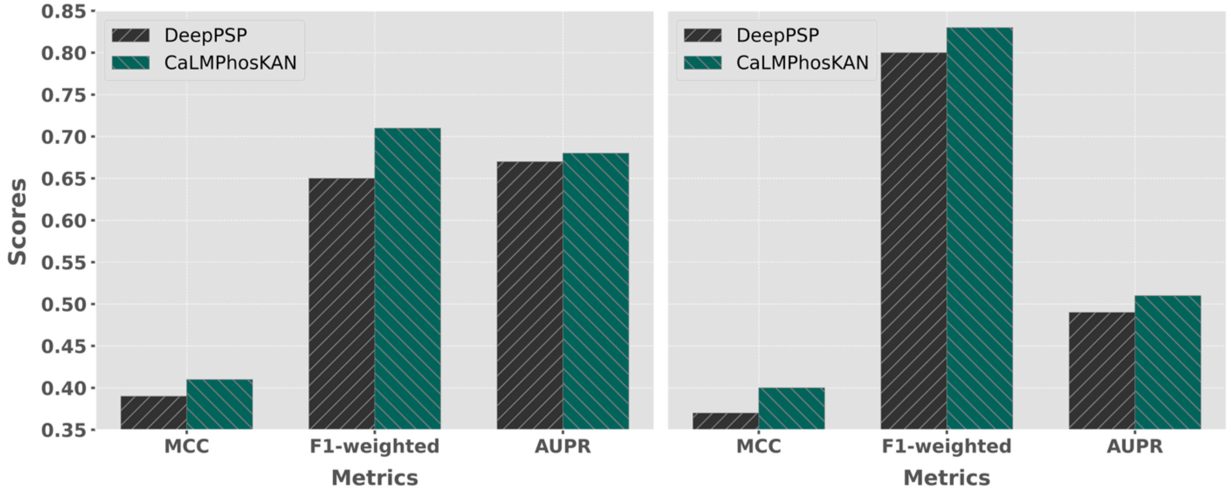

Figure S15.1. Bar graphs comparing the performance of CaLMPhosKAN and DeepPSP based on MCC, F1, and AUPR for **(a)** IDR regions (left) and **(b)** non-IDR regions (right) of proteins in the test set.

We also plotted the probability distribution of these models on the regions split by ground truth (P vs NP) using violin plots presented in Figure S15.2. For disordered regions, there is considerable overlap between P and NP sites. The distribution of P sites shows a wide range of scores, indicating variability and some uncertainty in predictions. Similar to DeepPSP, CaLMPhosKAN also shows considerable overlap between NP and P predictions, but the distribution for P sites is somewhat more concentrated than in DeepPSP. For non-disordered regions, in DeepPSP, the NP and P distributions are more distinct than in disordered regions, though there is still overlap. Similarly, in CaLMPhosKAN, the overlap between NP and P predictions is less pronounced compared to disordered regions. Predictions for P-sites are more concentrated towards higher scores, indicating greater confidence in these predictions. Overall, CaLMPhosKAN generally shows more concentrated predictions for P sites in both regions compared to DeepPSP, suggesting higher confidence. Both models, however, present a broader spectrum of predictions for P sites in disordered regions, highlighting the complexity of these areas.

Quantitative assessment of the overlap between P and NP predictions using Equation 4 further supports these observations. In non-disordered regions, CaLMPhosKAN shows a slightly better performance with less overlap (1.599) compared to DeepPSP (1.622). Conversely, in disordered regions, the overlap in CaLMPhosKAN is marginally less than in DeepPSP (1.619 vs 1.621). The derivation of Equation 4 is provided below.

Let $P_{X,R}$ represent the set of predictions for P-sites for model X in region R, and $N_{X,\,R}$ represent the set of predictions for NP-sites for model X in region R. Let $|P_{X,\,R}|$ be the number of positive predictions for model X in region R and $|N_{X,\,R}|$ be the number of negative predictions for model X in region R.

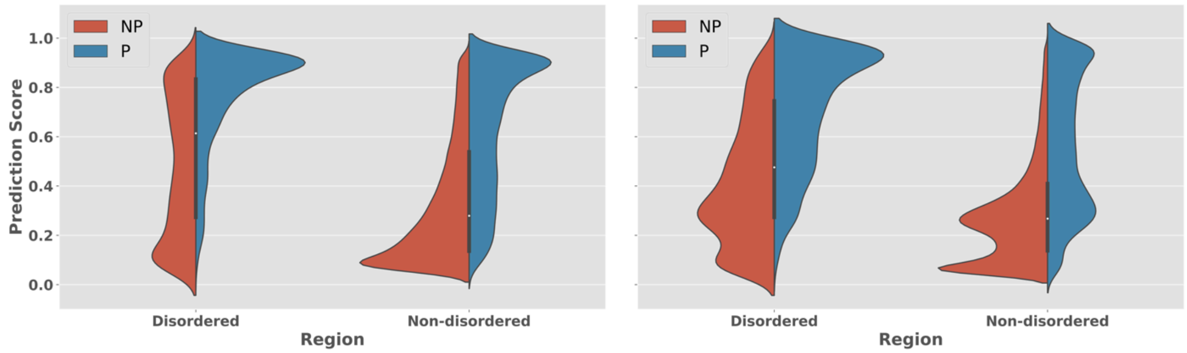

(a) **(b)**

Figure S15.2. Violin plots showing the distribution of prediction probabilities in disordered and non-disordered regions split by ground truth (P vs NP) for **(a)** DeepPSP (left) and **(b)** CaLMPhosKAN (right). The ground truth P is shown in blue while NP in red. The prediction score is sigmoidal and hence ranges from 0.0 to 1.0.

For each positive prediction $p \in P_{X,R}$, count the number of negative predictions $n \in N_{X,\ R}$ that are less than or equal to p. Let $C_{P,\ X,\ R}$ be this count which can be expressed as:

$$C_{P,X,R} \ = \ \sum_{p \in P_{X,R}} \sum_{n \in N_{X,R}} \Pi(n \leq p) \tag{1}$$

Now, for each negative prediction $n \in N_{X,R}$, count the number of positive predictions $p \in P_{X,\ R}$ that are less than or equal to n. Let $C_{NP,\ X,\ R}$ be this count which is expressed as :

$$C_{NP,X,R} \ = \ \sum_{n \in N_{X,R}} \sum_{p \in P_{X,R}} \Pi(n \geq p) \tag{2}$$

The total overlap *Overlap(X, R)* is the sum of the overlaps from positive and negative predictions normalized by the product of the number of positive and negative predictions:

$$overlap(X,\ R) \ = \ \frac{C_{P,X,R} + C_{NP,X,R}}{|P_{X,R}| + |N_{X,R}|} \tag{3}$$

Finally, the general equation of overlap between positive and negative distributions for any model X in any region R is obtained by combining equations (1), (2), and (3) :

$$overlap(X,\ R) \ = \ \frac{\sum_{p \in P_{X,R}} \sum_{n \in N_{X,R}} \Pi(n \leq p) + \sum_{n \in N_{X,R}} \sum_{p \in P_{X,R}} \Pi(p \geq n)}{|P_{X,R}| + |N_{X,R}|} \tag{4}$$

By using the above equation, the overlap for DeepPSP and CaLMPhosKAN is given below:

Predictor	Non-Disordered	Ordered
**DeepPSP**	1.622213	1.621862
**CaLMPhosKAN**	1.599573	1.619446

References

[1] H. D. Ismail et al. Rf-phos: A novel general phosphorylation site prediction tool based on random forest. BioMed research international, 2016(1):3281590, 2016.

[2] F. Zhou et al. Gps: a novel group-based phosphorylation predicting and scoring method. Biochemical and biophysical research communications, 325(4):1443–1448, 2004.

[3] J. Gao et al. Musite, a tool for global prediction of general and kinase-specific phosphorylation sites. Molecular & Cellular Proteomics, 12(9):2586-2600, 2010.

[4] D. Wang et al. Musitedeep: a deep-learning based webserver for protein post-translational modification site prediction and visualization. Nucleic Acids Research, 48(W1):W140– W146, 2020.

[5] S. Wang et al. A novel capsule network with attention routing to identify prokaryote phosphorylation sites. Biomolecules, 12(12):1854, 2022.

[6] F. Luo et al. DeepPhos: prediction of protein phosphorylation sites with deep learning, Bioinformatics, 35(16):2766–2773, 2019.

[7] X. Wang et al. TransPhos: A Deep-Learning Model for General Phosphorylation Site Prediction Based on Transformer-Encoder Architecture. International Journal of Molecular Sciences, 23(8):4263, 2022.

[8] T. Song et al. Attenphos: General Phosphorylation Site Prediction Model Based on Attention Mechanism. International Journal of Molecular Sciences, 25(3):1526, 2024.

[9] L. Guo et al. Deeppsp: a global–local information- based deep neural network for the prediction of protein phosphorylation sites. Journal of Proteome Research,20(1):346–356, 2020.

[10] S. C. Pakhrin et al. Lmphossite: a deep learning-based approach for general protein phosphorylation site prediction using embeddings from the local window sequence and pretrained protein language model. Journal of proteome research, 22(8):2548–2557, 2023.

[11] Raschka, S. (2018). Model Evaluation, Model Selection, and Algorithm Selection in Machine Learning. ArXiv. /abs/1811.12808.

[12] L. M. Iakoucheva *et al.*, 'The importance of intrinsic disorder for protein phosphorylation', *Nucleic Acids Res.*, vol. 32, no. 3, pp. 1037–1049, Feb. 2004, doi: 10.1093/nar/gkh253.

[13] G. Hu *et al.*, 'flDPnn: Accurate intrinsic disorder prediction with putative propensities of disorder functions', *Nat. Commun.*, vol. 12, no. 1, Art. no. 1, Jul. 2021, doi: 10.1038/s41467-021-24773-7.

[14] Thapa, N. et al., 'A deep learning based approach for prediction of *Chlamydomonas reinhardtii* phosphorylation sites', *Sci Rep.* **11**, 12550 (2021).

[15] Hao Lv, Fu-Ying Dao, Hasan Zulfiqar, Hao Lin, DeepIPs: comprehensive assessment and computational identification of phosphorylation sites of SARS-CoV-2 infection using a deep learning-based approach, Briefings in Bioinformatics, Volume 22, Issue 6, November 2021, bbab244.

[16] Wang, H. et al. The global phosphoproteome of Chlamydomonas reinhardtii reveals complex organellar phosphorylation in the flagella and thylakoid membrane. Mol Cell Proteomics 13, 2337–2353.

[17] Goodstein, David M., et al. "Phytozome: A Comparative Platform for Green Plant Genomics." Nucleic Acids Research, vol. 40, no. D1, 2012, pp. D1178-D118.
